# Characteristics of Resting-State Electroencephalogram Network in α-Band of Table Tennis Athletes

**DOI:** 10.3390/brainsci14030222

**Published:** 2024-02-27

**Authors:** Jilong Shi, Fatima A. Nasrallah, Xuechen Mao, Qin Huang, Jun Pan, Anmin Li

**Affiliations:** 1 School of Psychology, Shanghai University of Sport, Shanghai 200438, China; shijilong096@163.com (J.S.); xuechenmao.asprin@outlook.com (X.M.); annnny147@163.com (Q.H.); yanjunp@gmail.com (J.P.); 2 Center for Exercise and Brain Science, Shanghai University of Sport, Shanghai 200438, China; 3 Queensland Brain Institute, The University of Queensland, Saint Lucia, QLD 4067, Australia; f.nasrallah@uq.edu.au

**Keywords:** resting state, electroencephalogram, α-band, network characteristics, table tennis athlete

## Abstract

Background: Table tennis athletes have been extensively studied for their cognitive processing advantages and brain plasticity. However, limited research has focused on the resting-state function of their brains. This study aims to investigate the network characteristics of the resting-state electroencephalogram in table tennis athletes and identify specific brain network biomarkers. Methods: A total of 48 healthy right-handed college students participated in this study, including 24 table tennis athletes and 24 controls with no exercise experience. Electroencephalogram data were collected using a 64-conductive active electrode system during eyes-closed resting conditions. The analysis involved examining the average power spectral density and constructing brain functional networks using the weighted phase-lag index. Network topological characteristics were then calculated. Results: The results revealed that table tennis athletes exhibited significantly higher average power spectral density in the α band compared to the control group. Moreover, athletes not only demonstrated stronger functional connections, but they also exhibited enhanced transmission efficiency in the brain network, particularly at the local level. Additionally, a lateralization effect was observed, with more potent interconnected hubs identified in the left hemisphere of the athletes’ brain. Conclusions: Our findings imply that the α band may be uniquely associated with table tennis athletes and their motor skills. The brain network characteristics of athletes during the resting state are worth further attention to gain a better understanding of adaptability of and changes in their brains during training and competition.

## 1. Introduction

Table tennis is a fast-paced, high-precision sport necessitating swift reflexes, superior hand–eye coordination, and strategic decision-making skills [1]. Athletes must respond to their opponents’ moves with remarkable alacrity, making instantaneous decisions on returning the ball with both precision and power [2,3]. Over the years, researchers have extensively studied the cognitive processing characteristics of the brains of table tennis athletes (TTAs). They found that TTAs not only have advantages in perceptual [4], memory [3], and imaginative [5] cognitive processing, but also exhibit heightened functional and structural brain plasticity [6,7]. Despite extensive research on the cognitive processing characteristics of TTAs, investigations into the potential connectivity between different regions of the brain during rest have been limited.

Resting-state brain function is a vital physiological indicator of the body’s “baseline” state, reflecting the participant’s ability to perform cognitive and sensory–motor tasks and serving as a reliable biological marker [8]. During the resting state, the brain generates distinct frequency bands, including the α, β, θ, and δ bands, with the α band standing out as one of the most prominent. The α band primarily originates in the posterior regions of the brain’s cortex, including the temporal, parietal, and occipital lobes, and plays a crucial role in brain function and psychological states [9].

On one hand, the generation and regulation of the α band are associated with the excitatory and inhibitory processes of the cerebral cortex, reflecting the stability and equilibrium of the brain [9]. On the other hand, the intensity and frequency of the α band are linked to various cognitive processes, including attention [10], learning [11], memory [12], and emotion [13]. Additionally, the α band is associated with physical health [14,15], relaxation [16], and meditation [17]. In the eyes-closed resting state, the dominant frequency band with α frequency (about 8–13 Hz) is considered an important predictor of the effectiveness of cortical information processing during cognitive and sensory–motor activities [18]. Furthermore, the α band may be associated with motor performance in sports experts [18]. Studies have shown that the α band in the brains of TTAs during rest is more pronounced than in non-athletes, indicating their inclination for relaxation and concentration and thereby contributing to enhanced cognitive processing abilities and performance in competitions [5]. Discrepancies in the α band are frequently observed between novice and expert athletes, and these differences may serve as predictors of their peak performance [19]. Therefore, the α band in the brain during rest serves as an important indicator for assessing the cognitive processing abilities of TTAs [20]. The α band is typically divided into two subgroups: a “slow” range (α1, 8.0–10.5 Hz) and a “fast” range (α2, 10.5–13 Hz) [21]. The α1 band shows widespread attenuation across the cortex and is considered an indicator of non-specific attentional and expectancy processes [19]. In contrast, the α2 band reflects task-specific demands in the parieto-occipital regions within the somatosensory cortex [22]. Importantly, the α1 and α2 bands serve distinct roles in the brains of athletes [19,23].

However, research also suggests the presence of “neural efficiency” among sports experts [24]. The “neural efficiency” hypothesis posits that the energy metabolism rate in the cortical regions of the brain is paradoxically lower during cognitive tasks [25]. Moreover, populations with higher “neural efficiency” show more efficient and streamlined connections between brain neurons [25]. In a functional magnetic resonance imaging (fMRI) study focused on visuo–spatial tasks, TTAs exhibited decreased activation in the bilateral middle frontal gyrus, lingual gyrus, right middle orbitofrontal area, supplementary motor area, paracentral lobule, precuneus, angular gyrus, left supramarginal gyrus, inferior temporal gyrus, middle temporal gyrus, and cerebellum crus [6]. These findings suggest the presence of a more effective neural efficiency in TTAs. However, the assessment of neural efficiency is task-specific and it remains unknown whether TTAs also exhibit this effect during the resting state. Furthermore, some studies have emphasized that a reduction in activation should not necessarily be interpreted as an improvement in neural functional “efficiency” [26]. The brain connectome method provides an alternative perspective on the information transmission cost within the network for athletes [27,28]. This approach is valuable not only for understanding cognitive functions but also for extracting biomarkers capable of distinguishing between brains across diverse populations. Utilizing network metrics to characterize the classification feature space enhances the detection of mental states in athletes [28]. Additionally, the functional connectivity pattern of the brain network may vary based on various population characteristics, suggesting that functional brain networks can serve as biomarkers for identifying the connection patterns in athletes’ brains [29].

Numerous studies have demonstrated that prolonged training can induce alterations in brain functional connectivity [30,31]. Therefore, with advancements in human brain connectomics, it has become evident that the human brain does not operate in isolation; rather, it exhibits close connections between different regions and operates in coordination through specific connection patterns with efficient network transmission characteristics [32]. Previous investigations into brain networks among TTAs have primarily emphasized functional connectivity and efficiency connectivity [33,34]. For instance, Li et al. (2023) [33] observed reduced static functional connectivity in the right middle temporal gyrus and left inferior parietal gyrus among individuals with TTAs. Conversely, they identified increased dynamic functional connectivity originating from the left inferior temporal gyrus towards the prefrontal cortex, notably in the left middle frontal gyrus, left superior frontal gyrus medial, and left superior frontal gyrus dorsolateral regions. In a separate investigation, Gao et al. (2023) [34] examined disparities in effective brain connectivity between racket sports athletes and the control group. Granger causality values within the middle occipital gyrus exhibited a linear progression from negative to positive, indicating a gradual “neural proficiency” in effective connectivity from the control group to student athletes and ultimately to elite athletes.

To date, several techniques are available for investigating the brain network, including electroencephalogram (EEG), magnetoencephalography, and fMRI. However, EEG technology is notable for its high time resolution, low cost, ease of acquisition, and rich physiological information. Previous studies solely relied on functional connectivity and effective connectivity to analyze the connection relationships between local brain regions. However, delving into the topological properties of the whole brain network has provided a deeper understanding of this connectivity [33,34,35]. The examination of whole-brain topological properties unveils the overall structure and characteristics of the brain network, along with the connection patterns and topological features among diverse brain regions. This holistic perspective significantly contributes to comprehending the fundamental organizational principles and functional distribution of the brain [32,35]. For example, using betweenness centrality to search for specific brain hub nodes unique to TTAs, investigating differences in brain transmission efficiency between TTAs and the control group, observing the “rich-club” effect, and so forth. These insights transcend the capabilities of functional connectivity and effective connectivity alone [32]. Therefore, given the distinctive patterns of brain connectivity observed in athletes, as demonstrated not only by findings from fMRI studies but also by the significantly higher α band amplitudes observed in TTAs [5], and considering that heightened α band activity may signify potentially enhanced neural efficiency within the brain [24], we posit that TTAs not only display markedly enhanced functional connectivity strength relative to non-athletes but also exhibit superior information transmission efficiency within the brain’s network topological characteristics. Therefore, this study aims to utilize the method of whole-brain network topological properties to describe the specific static EEG network characteristics of TTAs, identify unique brain network biomarkers related to TTAs, and elucidate the functional features of the brain network in TTAs.

## 2. Materials and Methods

### 2.1. Subjects and Electroencephalogram Acquisition

We recruited a total of 48 participants and divided them into two groups. The first group comprised 24 high-level TTAs (12 females) from China Table Tennis College, Shanghai University of Sport, with an average age of 20.88 ± 2.15 years and an average training experience of 10.29 ± 3.39 years. All athletes in this group were nationally ranked at the first level within China’s sports system. The second group, serving as the control group, comprised 24 non-athletes (12 females) from Shanghai University of Sport, with an average age of 20.54 ± 2.16 years. We assessed their background through direct verbal communication, inquiring as to whether they had any history of sports training. Additionally, we used the questionnaires to assess participants’ sports experience, confirming that they had no prior experience in professional sports training and lacked any significant history of sports exercise. Both groups included right-handed participants with no history of major brain diseases or head surgeries. The study protocol underwent review and approval by the University Ethics Committee. The experiment was conducted in accordance with the ethical standards outlined in the Declaration of Helsinki. Participants volunteered to participate, fully understood the experimental procedures and objectives, and provided written informed consent.

Participants were seated on soft and comfortable chairs and were informed about the purpose of the experiment. They were instructed to relax without falling asleep. EEG data were collected during the resting state with participants’ eyes closed for a duration of 210 s. The EEG signals were recorded using active electrodes in Brain Vision Recorder (actiCHamp), version 2.0 (Brain Products GmbH, Gilching, Germany), where a 64-conductor Ag/AgCl electrode cap (actiCAP slim electrode) was placed on the scalp following the international 10-10 system. Horizontal and vertical EEGs were recorded. The horizontal EEG electrode was attached laterally to the right eye, and the vertical EEG electrode was attached inferiorly to the left eye. EEG signals were digitalized at a sampling rate of 1000 Hz with a band-pass filter of 0.01–100 Hz. The online reference electrode was set to FCz, and the ground electrode was placed at AFz. For offline re-referencing, one electrode was placed on the left mastoid, and another was placed on the right mastoid. The vertical electrooculogram was recorded below the left eye, and the horizontal electrooculogram was recorded at the outer canthus of the right eye. Electrode impedances were kept below 5 kΩ throughout the experiment. Participants’ behavior and the quality of the EEG signal were continuously monitored in real-time to ensure their alertness level was maintained. If any variations in the participants’ EEG patterns were detected due to factors such as coughing, manual movements, or signs of drowsiness, verbal reminders were given to ensure their alert state.

### 2.2. Data Preprocessing

EEG data were analyzed offline using a toolbox developed and based on the MATLAB 2014 platform, utilizing EEGLAB 14 (https://sccn.ucsd.edu/eeglab/index.php) software. The Standard-10-5-cap385 file from EEGLAB was utilized for electrode localization. Initially, a whole-brain average reference was adopted to re-reference the data, and the FCz electrode was restored. Unnecessary electrodes, including electrooculography (EOG) and bilateral mastoids, were removed. Subsequently, the continuous data underwent high-pass filtering at 0.1 Hz and low-pass filtering at 50 Hz using windowed-sinc FIR filters with a Hamming window through the FIRfilt plugin of EEGLAB (developed by A. Widmann: “www.unileipzig.de/~biocog/content/widmann/eeglab-plugins/” accessed on 13 April 2023). Notch filters at 48 Hz and 52 Hz were applied to mitigate power line interference. The data were segmented into 2-s segments. Subsequently, independent component analysis (ICA) was applied to identify and remove EEG artifacts associated with eye-blinks, muscle activity, cardiac signals, and line noise sources [36]. Finally, data segments with absolute voltage amplitude values exceeding 75 μV were discarded.

### 2.3. Resting-State EEG Power Spectral Density (PSD) Analysis

The power spectra of different frequency bands were calculated using the STUDY module of EEGLAB 14, utilizing fast Fourier transform (FFT) [15,37,38]. In the PSD calculation, the input signal was divided into small segments, and each segment was windowed to reduce spectral leakage. The small segments used for PSD calculation had a duration of 2 s each. These segments were chosen to strike a balance between capturing meaningful data and providing sufficient temporal resolution. A Hanning window was applied to these segments before FFT to mitigate spectral leakage and improve spectral estimation. FFT was then applied to each windowed segment to obtain the frequency representation of the signal. The squared magnitude of the FFT result represents the power at each frequency bin. Power was computed for all segmented data sections and averaged across all frequencies and epochs to obtain the mean power at each frequency point. Power was also independently calculated for each electrode channel to obtain power estimates at each frequency point on that channel. The PSDs were averaged across all electrode channels to obtain an overall PSD estimate, reflecting the brain’s activity levels at different frequencies. Subsequently, statistical analysis involved conducting two-sample *t*-tests on the overall average PSD for each individual in the two groups. After identifying significant differences in the average PSD between the two groups, the EEG signals were further divided into two frequency bands: α1 (8–10.5 Hz) and α2 (10.5–13 Hz). Statistical analysis was performed using MATLAB 2014, utilizing an independent samples *t*-test with a significance level set at *p* < 0.05.

### 2.4. Functional Coupling of Electroencephalogram Signals Based on Weighted Phase-Lag Index

Prior to constructing the brain’s functional network, source localization calculations were performed on resting-state EEG data from each participant using the default parameters of the CSD toolbox 1.1 (https://psychophysiology.cpmc.columbia.edu/Software/CSDtoolbox/index.html) to estimate current source density [39]. Based on the significant differences observed in the whole-brain average PSD, the selected frequency bands (α1 and α2) were utilized to construct the brain’s functional network using the weighted phase-lag index (wPLI) [40]. The wPLI is a widely used measure of functional connectivity in EEG and magneto-encephalography studies. It quantifies the connectivity between two brain regions by considering the phase differences between signals recorded from different sensors or electrodes, while incorporating the relative power of the signals as a weighting factor. This characteristic enables wPLI to be robust to the effects of volume conduction and to be capable of detecting nonlinear interactions between brain regions [40]. In comparison to other connectivity measures such as coherence and phase-lag index [41], the wPLI has proven effective in characterizing functional connectivity in EEG data, particularly in source-space analysis. It has been extensively used in studies exploring various neurological and psychiatric disorders [42], as well as investigating cognitive processes such as attention and memory [43]. We assessed functional connectivity using the wPLI as the metric.

Throughout the wPLI calculation process, we computed the wPLI for each electrode pair within every participant’s epoch. Subsequently, we averaged the wPLI values across all epochs to derive the mean wPLI for each electrode pair, thereby constructing the wPLI functional connectivity matrix for each participant’s brain. To evaluate the significance of connectivity differences between the two groups, we conducted permutation tests, which account for the non-parametric nature of our data. The specific details of the tests are as follows: (1) We conducted 1000 permutations to create a distribution of wPLI values under the null hypothesis, where there is no group difference. (2) We calculated the actual wPLI values between groups. (3) We compared the observed wPLI values with the null distribution to determine their significance level, resulting in a *p*-value for each connection. (4) The network-based statistics (NBS) method was employed to test the differences in functional connectivity strengths between 60 pairs of electrodes in each frequency band between the two groups of participants. The significance level was set at *p* < 0.05.

(1) $$wPLI=\frac{|\langle I(X)\rangle |}{\langle |I(X)|\rangle }=\frac{|\langle |I(X)|sign(I(X))\rangle |}{\langle |I(X)|\rangle }$$

I(X): This typically represents the complex-valued cross-spectral density or cross-power spectral density between two signals X. It captures the relationships between the phase and amplitude of the signals.

〈〉: These brackets denote the average or expected value of the enclosed quantity.

| |: The vertical bars denote the absolute value of the enclosed quantity.

sign(I(X)): This function returns the sign of I(X), which is +1 if I(X) is positive and −1 if I(X) is negative.

The wPLI formula can be understood as a method to estimate the phase synchronization between two signals while accounting for the strength of their connectivity. It is a weighted measure because it takes into consideration both the phase information and the amplitude (or strength) of the connection. The division by the average of the absolute value of I(X) helps normalize the measure.

### 2.5. Analysis of EEG Brain Network Topology

In the analysis of network topology, we chose to utilize the α band within the frequency range of 8–13 Hz. For each participant, a functional coupling matrix based on the wPLI was obtained in the α band from EEG data collected during the eyes-closed resting state. The coupling matrix had a format of 60 × 60, representing channel × channel connections. Using graph theory, each electrode was considered as a node in the network, and the connection strength represented the weighted edge between nodes. The topology of the brain network graph was analyzed. Ten network sparsity thresholds ranging from 0.05 to 0.5 were selected to compare the whole brain network’s topological properties. Graph theory analysis was performed using the GRETNA 2.0 [44] toolbox in MATLAB 2014. The analyzed network topology properties included clustering coefficient, local efficiency, betweenness centrality, and rich club, which are commonly used in graph theory. Statistical analysis was conducted using the GRETNA 2.0 [44] toolbox in MATLAB 2014, with an independent samples *t*-test and a significance level set at *p* < 0.05.

## 3. Results

### 3.1. Resting-State EEG Power Spectral Density

**Figure 1:**
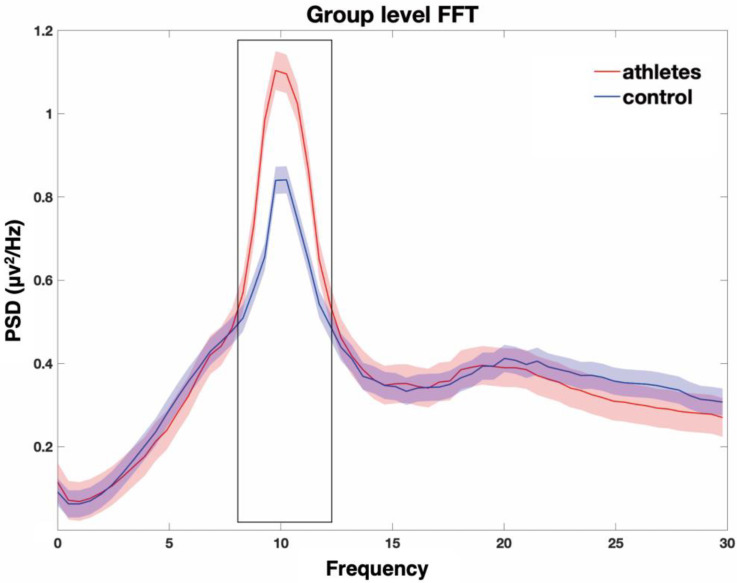
Average power spectral density. The difference in average PSD across the whole brain is shown, with the red line representing the TTAs and the blue line representing the control group. Red and blue shaded regions indicate the standard error, with significant difference highlighted by black boxes, *p* < 0.05.

**Figure 2:**
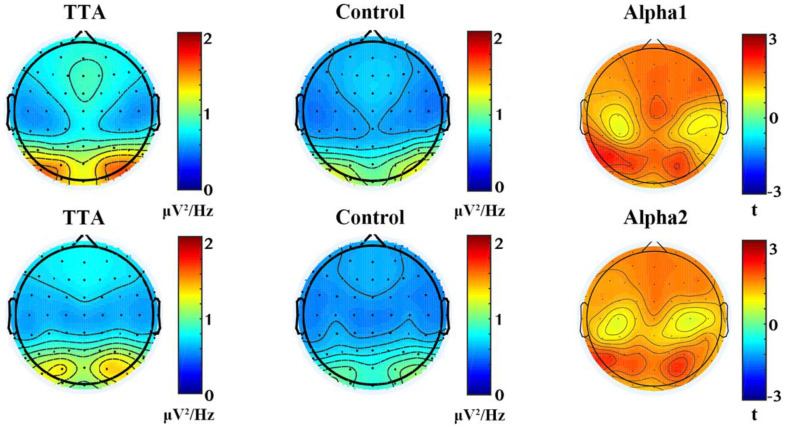
Power spectral density of α1 and α2. The top row shows the comparison between the TTAs and the control group in the α1 frequency band, while the bottom row shows the comparison in the α2 frequency band. The left column displays the frequency domain PSD plot for the athletes, the middle column shows the PSD plot for the control group, and the right column displays the results of the two-sample *t*-test, *p* < 0.05.

An independent samples *t*-test was utilized to compare the PSD between the TTA and the control group. The TTAs group exhibited higher PSD in the α band (8–13 Hz) compared to the control group, *p* < 0.05 (Figure 1). To further investigate the differences within the α band, it was divided into α1 (8–10.5 Hz) and α2 (10.5–13 Hz). In the α1 band, TTAs only exhibited significant differences at the P5 and P7 electrodes in the parietal lobe (*p* < 0.05) compared to the control group. In the α2 band, none of the electrodes showed a significant level of difference (Figure 2).

### 3.2. Resting-State Functional Connectivity

**Figure 3:**
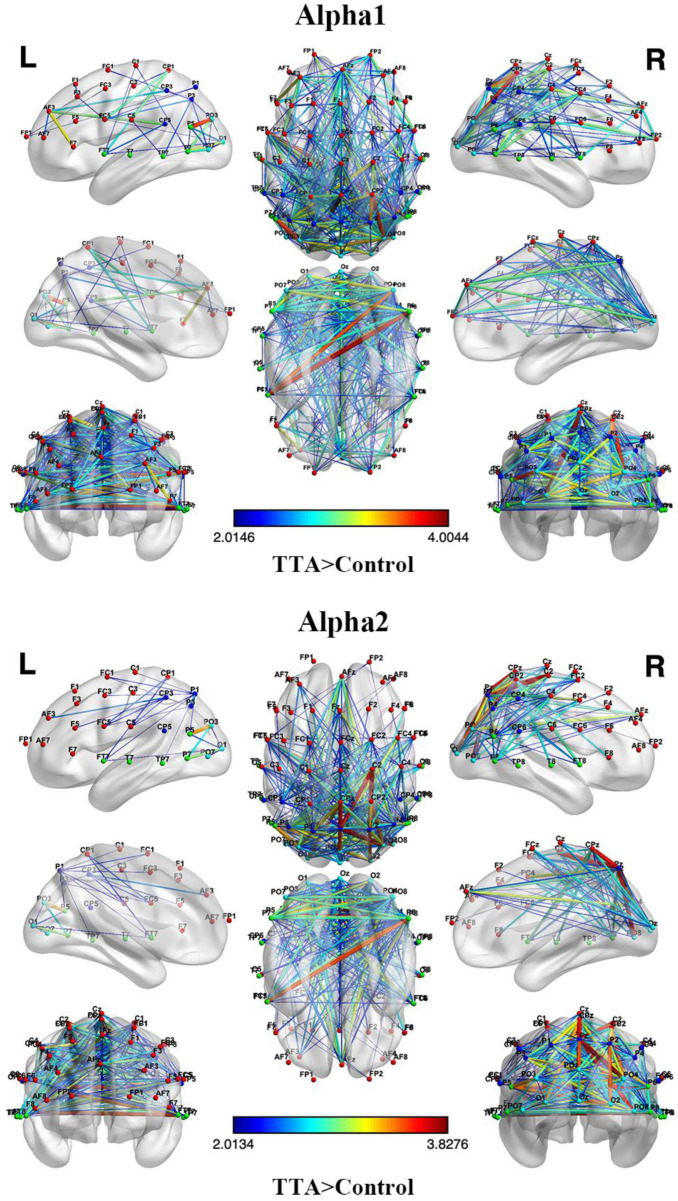
Functional connectivity of α1 and α2. The upper panel shows the functional connectivity differences between athletes and the control group in the α1 frequency band, while the lower panel shows the functional connectivity differences between athletes and the control group in the α2 frequency band. Lines connecting electrodes indicate functional connections, where athletes exhibit significantly greater connectivity than the control group; *p* < 0.05, NBS correction.

The analysis of resting-state functional connectivity revealed that the TTAs exhibited stronger functional connectivity compared to the control group in both the α1 and α2 bands. These findings remained statistically significant even after applying a more stringent NBS correction (*p* < 0.05). Specifically, in the α1 band, TTAs primarily showed stronger functional connectivity in the frontal, parietal, and occipital lobes. In the α2 band, TTAs mainly demonstrated stronger functional connectivity in the parietal and occipital lobes. Furthermore, compared to the control group, TTAs exhibited a higher number of significantly enhanced functional connections and higher connection strength in the α1 band compared to the α2 band (Figure 3).

### 3.3. Brain Network Hub Nodes

**Figure 4:**
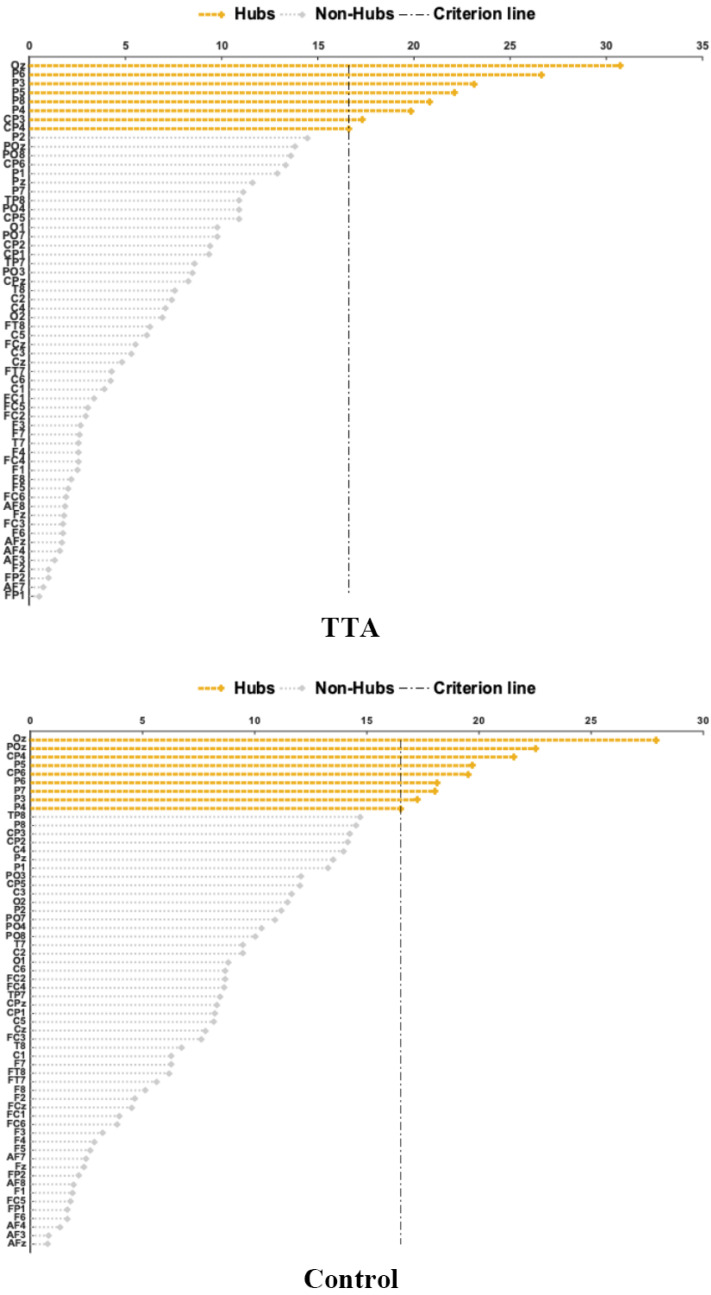
TTAs and control group brain network hub nodes. The upper side shows the hub nodes of the brain network in TTAs, while the lower side shows the hub nodes of the brain network in the control group.

The hub nodes in the α band of the brain network were identified using betweenness centrality for both the TTAs and the control group. Hub nodes refer to specific brain regions or nodes within a connectivity network that hold particular significance. These central nodes typically exhibit a higher degree (number of connections) or functional importance in the brain’s connectivity, often playing crucial roles in information transmission and integration [45]. It was observed that P3, P4, P5, P6, and CP4 were identified as hub nodes in both groups. Additionally, the TTAs exhibited additional hub nodes at P8 and CP3, while the control group had additional hub nodes at P7, CP6, and POz. Furthermore, the TTAs demonstrated a higher node degree compared to the control group (Figure 4).

### 3.4. Topological Properties of the Brain Network

**Figure 5:**
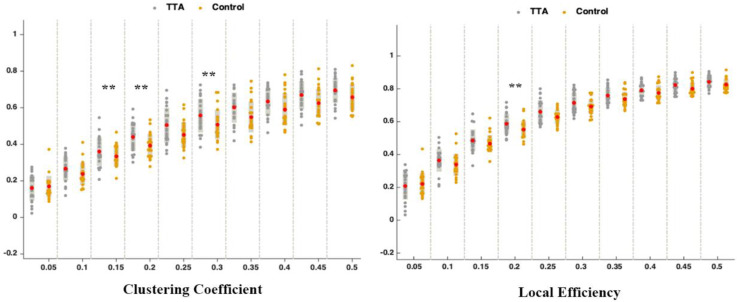
TTAs and control group differences in clustering coefficient and local efficiency. The *x*-axis represents the brain network sparsity, while the *y*-axis represents the values of clustering coefficient and local efficiency. Red dots indicate the average value. ** indicates the significance level, *p* < 0.05.

**Figure 6:**
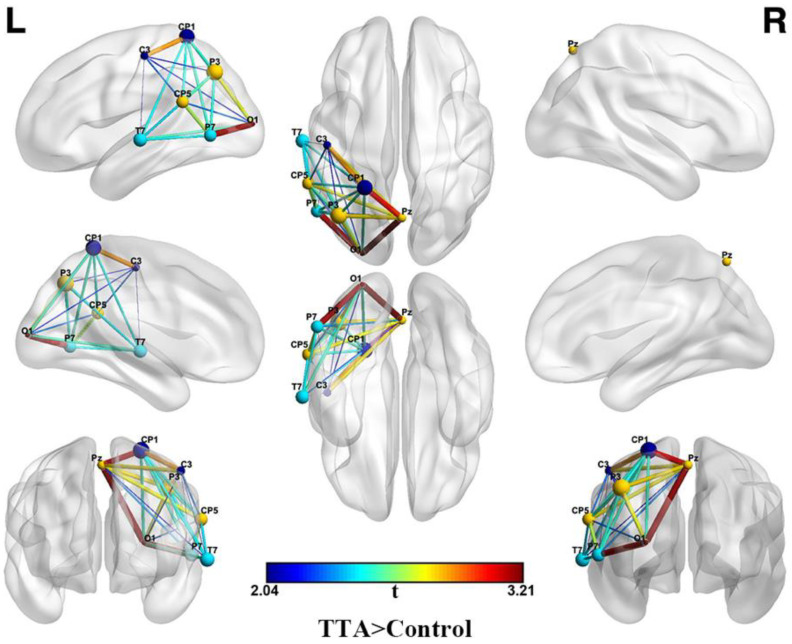
Rich club differences between TTAs and control group. The size and color of the node represent the significance level, while the thickness and color of the edge represent the strength of the functional connection. The significantly enhanced nodes and edges indicate the rich club differences formed; *p* < 0.05.

In the analysis of the topological properties of the brain network, it was found that, when the network sparsity was set at 0.2, the TTAs exhibited significantly stronger local efficiency and clustering coefficient compared to the control group (*p* < 0.05) (Figure 5). The clustering coefficient is a network analysis metric used to quantify the degree of connectivity between the nodes in a network, reflecting the extent to which a node’s neighbors are interconnected [32]. However, the clustering coefficient considers only direct connections among neighboring nodes, leading to the introduction of the concept of local efficiency [46]. Local efficiency measures the effectiveness of information transfer among neighbors of a specific node within a network, placing particular emphasis on the connections among a node’s immediate neighbors. Both the clustering coefficient and local efficiency measure the local information transmission capacity of a network [32]. This indicates that the TTAs had a more efficient and clustered brain network. Additionally, a stronger “rich club” phenomenon was observed on the left side of the brain in the TTAs (*p* < 0.05) (Figure 6). A “rich club” refers to a sub-set of highly connected nodes within a network that are densely interconnected with each other [47]. These nodes have a significantly higher number of connections compared to the average nodes in the network. In the context of the brain, the “rich club” includes highly interconnected regions that facilitate the transmission and integration of information across different brain areas.

## 4. Discussion

The expert–novice paradigm was employed in this study to compare the resting-state EEG data of highly skilled TTAs and a control group. By analyzing the differences in PSD, functional connectivity, and topological characteristics of the brain network during the resting state, our research hypotheses were tested and several noteworthy findings were identified. It is worth noting that this study provides the first examination of brain plasticity in TTAs through the lens of brain networks topological properties, providing valuable insights for future research. The subsequent sections will offer detailed explanations of the study’s findings.

### 4.1. The Power Spectral Density of Athletes and Non-Athletes

PSD analysis is a valuable tool for monitoring and identifying different cognitive states or tasks based on EEG signals [15,48]. For example, an increase in α band activity during a relaxed or resting state can indicate a person’s level of relaxation or focus [49]. Changes in PSD within specific frequency bands can offer valuable insights into various states, such as sleep stages, attention levels, and emotional states [50]. In this study, it was observed that TTAs displayed a notably higher global average PSD compared to the control group (Figure 1). This finding suggests that TTAs exhibit an increased energy distribution across the entire spectrum of brain electrical frequencies, distinguishing them from the control group. The increased global average PSD may reflect enhanced cognition- and motor-related electrical activities in the brains of TTAs [21]. Studies have shown that neural efficiency of sports experts’ brains is manifested in more effective neural network connections, faster reaction times, more precise coordination and motor skills, and improved sports memory [6,51,52]. Therefore, these differences may be attributed to the efficient utilization of cognitive resources, enhanced attentional control abilities, and adaptive neuroplastic changes in the brain among TTAs [6,53].

Previous research has indicated that elite karate athletes exhibit stronger α1 band amplitudes in the parietal and occipital lobes compared to amateur athletes and non-athletes [51]. However, in this study, we found that, in the α1 band, TTAs not only demonstrated stronger PSD in the parietal and occipital lobes but also exhibited enhanced PSD in the frontal lobe. This inconsistency may stem from variations in the classification of their specific sports disciplines, reflecting differences in the expression of fine motor skills and gross motor skills [54]. Table tennis, as a sport falling under fine motor skills, requires precise hand–eye coordination and accurate hand control [55]. The frontal lobe, closely associated with hand–eye coordination, also plays a crucial role in executing tasks demanding fine motor control [56]. In tasks requiring accurate target localization and movement execution, the frontal lobe integrates visual information and motor planning to ensure consistent coordination between the focus of the eyes and hand movements [57]. In the α2 band, differences were primarily observed in the occipital lobes (Figure 2). These regional PSD differences may be associated with the cognitive and attentional demands of table tennis. Given the crucial roles of the frontal and parietal lobes in cognitive tasks and attentional control [53], and the involvement of the occipital lobe in relaxation and perceptual processing, the heightened PSD in these regions among TTAs may indicate their strengths in attentional control [6], perceptual processing [4], and motor execution [58]. Although the differences in the α2 band were less prominent and not statistically significant, an increase in occipital PSD was still observed. This could be attributed to specific patterns of brain electrical activity exhibited by TTAs during states of relaxation and focus. Further research is needed to investigate the PSD differences in different brain regions within the α1 and α2 bands among TTAs and to gain a better understanding of the relationship between these differences and cognition, attention, and motor execution.

### 4.2. The Functional Connectivity between Athletes and Non-Athletes

Functional connectivity refers to the inter-relation and coordination between different brain regions, reflecting the transfer and collaboration of information to support various cognitive, perceptual, and motor functions [15,59]. This study revealed that TTAs exhibited heightened functional connectivity in specific brain regions compared to the control group, consistent with earlier findings [60]. Particularly in the α1 band, TTAs exhibited increased functional connectivity in the frontal, parietal, and occipital lobes, signifying enhanced information transfer and coordination among these regions (Figure 3). Similarly, in the α2 band, TTAs demonstrated heightened functional connectivity, mainly in the parietal and occipital lobes (Figure 3). Moreover, the disparities in functional connectivity were more pronounced in the α1 band compared to the α2 band. The α1 band is associated with relaxation, rest, and cognitive regulation [61], whereas table tennis demands rapid reactions, precise execution, and sustained attention [1]. The heightened functional connectivity observed in the α1 band among TTAs may be attributed to their superior attentional control and cognitive resource utilization [61], facilitating efficient information transfer and coordination between brain regions, thereby conferring advantages in cognition and motor execution [62]. In the α2 band, TTAs exhibited heightened functional connectivity, mainly in the parietal and occipital lobes, which is crucial for movement planning, execution, and sensory processing [1]. In another resting-state fMRI study, it was also noted that TTAs exhibited stronger functional connections compared to the control group [1]. Consequently, the intensified connections among these regions in TTAs may stem from extended practice and professional training, resulting in enhanced coordination and accuracy during motor execution [1,62]. Although a reduction in enhanced functional connectivity was observed in athletes in the α2 band compared to α1, localized enhancement of functional connectivity in specific regions may still have significant implications for the skills and performance of TTAs. In a study comparing the functional connectivity of shooting athletes and a control group, it was similarly found that athletes exhibited fewer enhanced functional connections in the α2 band compared to α1 [20]. While current research has explored these distinctions, further investigation is required to reveal the underlying neurophysiological mechanisms accounting for these differences between experts and novices.

### 4.3. The Network Topological Properties of Athletes and Non-Athletes

The analysis of network topological properties revealed significant differences between TTAs and the control group. Both groups exhibited hub nodes in the α band brain network, crucial for information integration and transmission. However, each group displayed a distinct set of hub nodes, indicating a diverse distribution of hub nodes in the α band brain networks between TTAs and the control group. These specific hub node distributions may mirror the impact of table tennis on the brain’s network structure, potentially linked to the unique cognitive and motor demands of the sport [63]. Notably, hub nodes were identified in the parietal lobe for both TTAs and the control group, corroborating prior research findings and underscoring their importance in information processing [64]. The functional significance of these specific hub nodes and their correlation with table tennis skills and cognitive abilities merit further investigation. Nonetheless, these findings offer valuable insights into the influence of table tennis on the α band brain network.

TTAs exhibited significantly higher local efficiency and clustering coefficients in the brain network compared to the control group. This suggests that table tennis training and experience positively impact the organization and function of the brain network. Local efficiency measures information transfer efficiency in a network by evaluating the tightness of connections between nodes and their neighbors [64]. The higher local efficiency observed in TTAs suggests that their brain networks are more efficient in information transfer and integration. The clustering coefficient, measuring the degree of clustering among nodes in a network, was also higher in TTAs. This indicates a more modular and clustered characteristic in the brain network of TTAs, facilitating efficient and coordinated information transfer between different brain regions. The higher clustering coefficient suggests that TTAs have stronger local connectivity and more clustered functional modules in their brain network [32]. The combination of higher local efficiency and clustering coefficient indicates that TTAs have enhanced local information transmission capacity in their brain networks. This finding suggests that the improved brain coordination in TTAs manifests in local aspects of the brain rather than in global network properties. This finding is also inconsistent with a study that only found higher local efficiency but not a higher clustering coefficient in the brain structural network of gymnasts [64]. This discrepancy may also be attributed to the differences in sports disciplines; gymnastics is a closed-skill sport, while table tennis represents an open-skill sport more susceptible to environmental influences. Improvements in local information processing efficiency may support improved response accuracy, which is crucial for success in table tennis.

Furthermore, a stronger “rich club” was observed in the left temporo-parietal-occipital lobe of the brain in TTAs, indicating a specific location where TTAs exhibit enhanced local functionality, and thus suggesting that TTAs have more connections and communication in this brain area [65]. This finding is consistent with the results of a resting-state fMRI investigation. The fMRI study similarly observed heightened dynamic functional connectivity in the left temporo-parietal lobe among TTAs [1]. Consequently, we posit that this outcome could be associated with the specific skills demanded by table tennis, including hand–eye coordination, spatial perception, and strategic planning. The presence of a more prominent “rich club” in this specific brain region emphasizes the specialized adaptations of the brain network in TTAs to meet the demands of table tennis. Additionally, these results suggest the presence of a lateralization effect [47]. In table tennis, athletes typically use a specific arm (left or right) to strike the ball, potentially leading to lateralization effects in the brain during movement-related tasks. Specifically, right-handed athletes may exhibit stronger functional connections in the left hemisphere, while left-handed athletes may show stronger functional connections in the right hemisphere. It is worth noting that both the TTAs and the control group in this study were right-handed, potentially contributing to the observed lateralization effect [66].

### 4.4. Limitations

This study does have certain limitations. Firstly, it was conducted with a relatively small sample size and included only right-handed participants. Further research, involving a larger and more diverse sample size, including athletes with varying dominant hand preferences, is needed to validate and explore the presence and impact of lateralization effects in TTAs. Secondly, in terms of eye status and frequency band selection, this study solely analyzed data from the eyes-closed resting state. This choice was motivated by the prominent α band activity typically observed during eyes-closed resting-state EEG. Nonetheless, recent research has underscored disparities in brain functional connectivity between eyes-open and eyes-closed states [67,68]. Furthermore, the exclusive focus on the α band represents a constraint, notwithstanding prior findings suggesting α band distinctions between experts [18,19] and novices and the disparities in average PSD observed between the TTAs and control groups in this study. However, we acknowledge the necessity of analyzing multiple frequency bands. Subsequent research endeavors should incorporate eyes-open states and explore additional frequency bands for comprehensive analysis. Finally, it is important to note that physical exercise itself can induce changes in brain plasticity. Therefore, further investigation is required to determine if the observed brain plasticity is specifically attributed to table tennis training. Future research could incorporate a control group consisting of athletes from different sports to compare and substantiate the findings of this study. Despite these limitations, this study represents the inaugural exploration of the resting-state brain network topological properties in TTAs, providing valuable preliminary insights and establishing the groundwork for further investigations in this domain.

## 5. Conclusions

This study unveiled the distinctive network characteristics of TTAs in the α band. The findings indicate that TTAs demonstrate stronger functional connections and higher transmission efficiency within the α band brain network, particularly at the local level. Furthermore, these local transmission enhancements manifest in a lateralization effect, with more potent and interconnected hubs observed in the left hemisphere of TTAs’ brains. These results suggest that the unique characteristics of the α band brain function network of TTAs, presumably shaped by extensive training and inherent individual differences, may contribute to their remarkable motor skill performance. In summary, gaining a deeper understanding of the neural mechanisms that distinguish elite athletes from non-athletes not only provides a solid foundation for enhancing future training strategies but also highlights the potential of EEG and the brain connectome as biomarkers for detecting stages and levels of athletes’ training.

## Data Availability

The data set supporting the conclusions of this article will be made available by the authors on reasonable request. The data are not publicly available due to ongoing analysis and additional research projects that are building upon this dataset.
